# Etoricoxib-Cannabidiol Combo: Potential Role in Glioblastoma Treatment and Development of PLGA-Based Nanoparticles

**DOI:** 10.3390/pharmaceutics15082104

**Published:** 2023-08-09

**Authors:** Joanna Kuźmińska, Agnieszka Sobczak, Aleksandra Majchrzak-Celińska, Izabela Żółnowska, Aleksandra Gostyńska, Barbara Jadach, Violetta Krajka-Kuźniak, Anna Jelińska, Maciej Stawny

**Affiliations:** 1 Chair and Department of Pharmaceutical Chemistry, Poznan University of Medical Sciences, Grunwaldzka 6, 60-780 Poznań, Poland; 2 Doctoral School, Poznan University of Medical Sciences, Bukowska 70, 60-812 Poznań, Poland; 3 Chair and Department of Pharmaceutical Biochemistry, Poznan University of Medical Sciences, Święcickiego 4, 60-781 Poznań, Poland; 4 Chair and Department of Pharmaceutical Technology, Poznan University of Medical Sciences, Grunwaldzka 6, 60-780 Poznań, Poland

**Keywords:** etoricoxib, cannabidiol, glioblastoma, PLGA, nanocarriers, drug nanoformulations

## Abstract

Background: Glioblastoma (GBM) is the most frequently occurring primary malignant central nervous system tumor, with a poor prognosis and median survival below two years. Administration of a combination of non-steroidal anti-inflammatory drugs and natural compounds that exhibit a curative or prophylactic effect in cancer is a new approach to GBM treatment. This study aimed to investigate the synergistic antitumor activity of etoricoxib (ETO) and cannabidiol (CBD) in a GBM cell line model, and to develop poly(lactic-co-glycolic acid) (PLGA)-based nanoparticles (NPs) for these two substances. Methods: The activity of ETO+CBD was determined using the MTT test, cell-cycle distribution assay, and apoptosis analysis using two GBM cell lines, namely, T98G and U-138 MG. The PLGA-based NPs were developed using the emulsification and solvent evaporation method. Their physicochemical properties, such as shape, size, entrapment efficiency (EE%), in vitro drug release, and quality attributes, were determined using scanning electron microscopy, diffraction light scattering, high-performance liquid chromatography, infrared spectroscopy, and differential scanning calorimetry. Results: The combination of ETO and CBD reduced the viability of cells in a dose-dependent manner and induced apoptosis in both tested GBM cell lines. The developed method allowed for the preparation of ETO+CBD-NPs with a spherical shape, mean particle size (MPS) below 400 nm, zeta potential (ZP) values from −11 to −17.4 mV, polydispersity index (PDI) values in the range from 0.029 to 0.256, and sufficient EE% of both drugs (78.43% for CBD, 10.94% for ETO). Conclusions: The combination of ETO and CBD is a promising adjuvant therapeutic in the treatment of GBM, and the prepared ETO+CBD-NPs exhibit a high potential for further pharmaceutical formulation development.

## 1. Introduction

The inflammation process is associated with the development and progression of cancer [1,2,3]. It reduces the immune system response and, therefore, affects the chemotherapeutic agents’ sensitivity. The continuous secretion of inflammatory cells, growth factors, and DNA-damage-promoting agents, including reactive oxygen species (ROS), induces tissue injury and DNA mutations, increasing the risk of carcinogenesis. A growing number of studies show that inflammation is crucial for glioblastoma (GBM) progression [1,2,3]. GBM is the most frequently occurring primary malignant central nervous system tumor [4]. It represents 57% of all gliomas and 48% of all primary malignant tumors in the brain [4]. It is characterized by extreme invasiveness and heterogeneity, as well as overexpression of the ABC transporters, complicating therapy [5,6,7,8]. The standard initial treatment approach is maximal safe surgical resection, resulting in a reduction in tumor volume, followed by radiotherapy with concomitant chemotherapy with temozolomide. Antimitotic therapy using low-intensity electric fields delivered by transducer arrays applied to the scalp during temozolomide treatment can also prolong patients’ overall survival [9,10]. However, despite the advances in current anticancer therapy, including surgery, radiotherapy, and chemotherapy, the prognosis of GBM remains poor, with a median survival of below two years. For this reason, the search for new treatment options for GBM is a high priority in the medical and pharmaceutical sciences [4].

GBM cells and inflammatory cells are phenotypically similar, since they produce cytokines and chemokines [3]. Hence, anti-inflammatory drugs, including non-steroidal anti-inflammatory drugs (NSAIDs), may interfere with the tumor microenvironment, increasing tumor-cell apoptosis and ameliorating the response to therapy. Inflammatory and neoplastic cells may undergo similar biochemical processes due to the activation of the NF-ĸB transcription factor. Activation of this factor stimulates the production of pro-inflammatory cytokines, metalloproteinases, and ROS, and it may cause tissue damage and DNA mutations. Interleukin 6 (IL-6) and growth factors in inflammatory and tumor cells can induce STAT3 activation, leading to cell proliferation and prolonged survival. Numerous studies show that NSAIDs increase apoptosis and sensitivity to conventional therapies and decrease invasion and metastasis. Some NSAIDs retain their anticancer effects even when they do not act as cyclooxygenase-2 (COX-2) inhibitors or act through their ability to inhibit drug-resistance molecules [1,2,3]. One of the promising candidates for NSAIDs in the treatment of cancer is etoricoxib (ETO). So far, the antitumor activity of ETO has been demonstrated in the treatment of lung [11,12,13,14,15,16], prostate [17,18], and colon cancers [19,20]. In this regard, our previous study showed that ETO exerts cytotoxicity and induces cell-cycle arrest and apoptosis in glioblastoma (GBM) cells. These actions are mediated via the inhibition of the Wnt/β-catenin and COX-2/PGE2/EP4 signaling pathways in GBM cells [21].

Novel trends in the development of cancer treatment strategies include the use of natural compounds with NSAIDs [22] or with conventional anticancer agents [23]. Natural products are accessible, inexpensive, and show little systemic toxicity [24]. Additionally, they have multiple mechanisms of action that can potentiate the outcomes of chemotherapeutics [23,25]. In this regard, one of the most promising natural compounds is cannabidiol (CBD), which is one of the main components of *Cannabis sativa* [26]. Importantly, unlike Δ9-tetrahydrocannabinol, CBD is characterized by a lack of psychotropic action [27]. CBD has a strong antiproliferative and pro-apoptotic effect against many different types of cancer, both in cultured cancer cell lines and in murine tumor models. The antitumor mechanisms differ depending on the type of tumor and may manifest as cell-cycle arrest, cell-death induction, or several parallel mechanisms. In addition, CBD may also inhibit tumor migration, invasion, and neovascularization. It is suggested that CBD not only acts on cancer cells but may also affect the tumor microenvironment—for example, by modulating infiltrating mesenchymal cells and immune cells [28]. Its role in GBM therapy remains to be elucidated.

Moreover, the challenge in the successful treatment of GBM is the effective delivery of the active substance to the tumor cells. Nanotechnology-based drug delivery systems, especially polymeric poly(lactic-co-glycolic acid) (PLGA)-based nanoparticles (NPs), are considered to be a forefront platform in drug delivery to the brain [29]. Encapsulating active compounds into such carriers allows penetration through the blood–brain barrier, protects the compounds from degrading factors, and masks the unfavorable properties of the active substances, such as high toxicity. Moreover, PLGA NPs ensure the sustained release of the drug and enhance the half-life of the drug in the circulatory system [30]. Due to their attractive properties, such as biodegradability and biocompatibility, protection of drugs from degradation, and the ability to deliver nanoparticles to specific organs or cells, PLGA NPs have attracted our attention. Importantly, PLGA has already been approved by the Food and Drug Administration (FDA) and the European Medicines Agency for use in drug delivery systems for parenteral administration [31]. Therefore, PLGA NPs could be further developed into an intravenous dosage form of ETO and CBD, thus contributing to overcoming the bioavailability limitations. Intravenous administration allows for bypassing the digestive system, avoiding first-pass metabolism and potential deactivation of the drug, which results in 100% bioavailability.

To the best of our knowledge, the incorporation of ETO and CBD into one nanoformulation has not yet been reported. Moreover, considering that both substances are characterized by low bioavailability and have been presented in numerous studies as potential drugs that could be used to treat cancer, our research seems justified. This research aimed to determine the synergistic antitumor activity of ETO and CBD in an in vitro model of GBM cell lines, and to develop PLGA-based NPs, enabling the effective incorporation of both of these substances into the polymeric matrix. The effects of ETO and CBD on survival, apoptotic cell death, and the distribution of the cell-cycle phases in GBM cells were assessed, and the PLGA-based NPs co-loaded with ETO and CBD were developed and characterized physicochemically.

## 2. Materials and Methods

Etoricoxib (ETO) was purchased from NOVACHEMISTRY, Unit11, Ark Business Centre, Gorden Road, Loughborough, LE11 1JP, United Kingdom; cannabidiol (CBD) was purchased from Medcolcanna Organics Inc., Distrito Especial, Colombia. Poly(D,L-lactide-*co*-glycolide) (PLGA, 50:50, molecular weight 7000–17,000), polyvinyl alcohol (PVA, molecular weight 30,000–70,000), and dialysis tubing cellulose membrane with an avg. flat width of 10 mm (0.4 in.) were obtained from Sigma-Aldrich, St. Louis, MO, USA. All organic solvents used in the studies were of analytical or high-performance liquid chromatographic grade.

Human glioblastoma WHO grade IV T98G (ATCC number: CRL-1690) and human glioblastoma grade IV UM-138 MG (ATCC number: HTB-16) cell lines were obtained from the European Collection of Authenticated Cell Cultures (ECACC) and the American Type Culture Collection (ATCC, Manassas, VA, USA), respectively.

### 2.1. In Vitro Studies

#### 2.1.1. Biological Assays

T98G and U-138 MG cells were cultured in ATCC-formulated Eagle’s Minimum Essential Medium supplemented with 10% fetal bovine serum and 1% antibiotic solution, at 37 °C, in a humidified 5% CO_2_ atmosphere. For the viability assay, 1 × 10^4^ cells/well were seeded in 96-well plates and incubated for 24 h. Then, the cells were treated with different concentrations (ranging from 1 to 100 μM) of ETO, CBD, or their combination. Cells treated with 0.1% dimethyl sulfoxide (DMSO) were used as the control.

#### 2.1.2. MTT Viability Assay

After 24 h of the treatment, the cell viability was assessed using the MTT (3-[4,5-dimethylthiazole-2-yl]-2,5-diphenyltetrazolium bromide) assay. The cells were washed twice with phosphate-buffered saline (PBS) and incubated with a medium containing 0.5 mg/mL MTT for 4 h. The formazan crystals formed by viable cells were dissolved in acidic isopropanol, and the absorbance was measured at 570 nm and 690 nm using a spectrophotometer.

#### 2.1.3. Cell-Cycle Distribution Analysis

For cell-cycle analysis, 1 × 10^5^ cells per well were seeded in 6-well plates and incubated for 24 h before treatment. The tested compounds (ETO, CBD, or their combination) were added to the cells, and they were further incubated for 24 h. Cells were harvested by trypsinization, fixed in ice-cold 70% ethanol, and stored at −20 °C until staining. After overnight storage, cells were stained with propidium iodide in the presence of RNase A and analyzed by flow cytometry on the Muse^®^ Cell Analyzer.

#### 2.1.4. Apoptosis Analysis

The assessment of apoptosis was performed by analyzing the externalization of phosphatidylserine using annexin V staining and 7-aminoactinomycin D (7-AAD) staining; 1 × 10^5^ cells per well were seeded in 6-well plates and incubated for 24 h before treatment. The tested compounds (ETO, CBD, or their combination) were added to the cells, and they were further incubated for 24 h. Cells were collected by trypsinization and stained with annexin V and 7-AAD solution. The cells were then analyzed by flow cytometry on the Muse^®^ Cell Analyzer.

The data obtained from the experiments were analyzed using the Muse^®^ 1.5 analysis software. The experiments were repeated three times, and four measurements were taken per assay. The results of this study would help in understanding the effects of ETO and CBD, both individually and in combination, on GBM cell viability, cell-cycle distribution, and apoptosis. These findings may have implications for potential therapeutic approaches in the treatment of GBM.

### 2.2. Synthesis of PLGA NPs

PLGA NPs were prepared using the solvent evaporation method. The organic phase for ETO, CBD, and ETO+CBD-loaded PLGA NPs was prepared by dissolving 5.0 mg of ETO, or 5.0 mg of CBD, or 2.5 mg of ETO and 2.5 mg of CBD in a previously prepared solution of 50.0 mg of PLGA in dichloromethane (2 mL), respectively. Then, the organic phase was emulsified by adding it to an aqueous phase consisting of a 2% (*w*/*v)* solution of PVA under probe sonication applied for 5 min at 40% voltage efficiency in cycling mode (30 s on and 30 s off). Then, the dichloromethane was evaporated on a magnetic stirrer for 20 h at room temperature. In order to remove the PVA solution, the samples were centrifuged and washed three times with deionized water. The resulting water suspensions of PLGA NPs were frozen at −40 °C, lyophilized in freeze-dryer (Epsilon 2–4 LSC plus, Martin Christ, Osterode am Harz, Germany) within 36 h at −35 °C, with a pressure of 0.2 mbar, before being stored at 4 °C. The compositions of the prepared PLGA NPs are presented in Table 1.

### 2.3. Characterization of NPs

#### 2.3.1. Mean Particle Size (MPS), Polydispersity Index (PDI), and Zeta Potential (ZP) Analysis

The MPS, PDI, and ZP of the developed PLGA NPs (blank-NPs, CBD-NPs, ETO-NPs, and ETO+CBD-NPs) were determined using the Malvern Zetasizer Nano ZS (Malvern Instruments, Malvern, UK). The dynamic light scattering (DLS) and laser Doppler electrophoresis (ELD) techniques were used for the determination of MPS and ZP, respectively; 100 μL of each PLGA NPs suspension was diluted with deionized water to 10 mL, vortexed, and transferred to polycarbonic cuvettes dedicated to the MPS, PDI, and ZP measurements.

#### 2.3.2. Differential Scanning Calorimetry (DSC) Analysis

The DSC studies of ETO, CBD, and lyophilized PLGA NPs (blank-NPs, ETO-NPs, CBD-NPs, and ETO+CBD-NPs) were performed using a Netzch 214 Plyma instrument equipped with an intercooler system; 5.0 mg of each tested sample was placed in sealed aluminum crucibles and heated from 25 °C to 180 °C under a nitrogen atmosphere. The scanning rate was 10 °C min^−1^.

#### 2.3.3. Fourier-Transform Infrared (FT-IR) Studies

FT-IR spectra were collected on an IRAffinity-IS Fourier-Transform Infrared Spectrophotometer (Shimadzu, Kyoto, Japan) instrument in the range of 400–4000 cm^−1^, with a resolution of 4.0 cm^−1^ and 40 scans.

First, 1 mg of lyophilized PLGA NPs (ETO-NPs, CBD-NPs, and ETO+CBD-NPs) was micronized and mixed with 300 mg of KBr (Sigma Aldrich, Saint Louis, MO, USA) to produce tablets (1.3 cm × 0.1 cm). The reference spectra were obtained by using the same tableting method, by mixing 1 mg of ETO, CBD, or blank-NPs with 300 mg of KBr. The obtained spectra of the loaded PLGA NPs were compared with the reference spectra, and the purity index (PI) indicating the degree of matching between the two spectra was determined for each pair. PI is expressed as the least-squares-fit coefficient calculated for every intensity pair of the two spectra being compared, and it was calculated using the following formula:

(1) $$PI=\frac{\sum_{i}^{n}(s_{i}-\bar{s})\times (r_{i}-\bar{r})}{\sqrt{\sum_{i}^{n}{\left(s_{i}-\bar{s}\right)}^{2}\times \sum_{i}^{n}{\left(r-\bar{r}\right)}^{2}}}$$

where *s_i_* and *r_i_* are the respective intensities for the same horizontal coordinate value, and n is the number of data points; $\bar{s}$ and $\bar{r}$ are the average intensities of each spectrum.

The purity value is between 0 and 1, where 0 indicates a lack of identity between the two spectra, and 1 indicates that the two spectra are identical.

#### 2.3.4. Scanning Electron Microscope Analysis

The morphology of the lyophilized PLGA NPs (blank, ETO-NPs, CBD-NPs, and ETO+CBD-NPs) was determined using a scanning electron microscope operated at an accelerated voltage of 5 kV. The lyophilized NPs were applied directly to the plate and analyzed using a Carl Zeiss EVO 40 scanning electron microscope.

#### 2.3.5. Drug Loading (DL%) and Entrapment Efficiency (EE%)

The DL% and EE% of ETO and CBD in loaded PLGA NPs were determined by the HPLC method. The chromatographic analysis was performed on an Agilent 1260 Infinity II LC System (Agilent Technologies, Bolinem, Germany) equipped with a quaternary pump (model G7111B) and degasser, a vial sampler (model G7129A) set at 15 °C ± 2 °C, a multicolumn thermostat (model G7116A) set at 30 °C ± 0.8 °C, and a diode array detector (DAD WR, model G7115A). The detection wavelength was adjusted to 230 nm and 235 nm for CBD and ETO, respectively. The separation between CBD and ETO was achieved on the reverse-phase column used as a stationary phase (InfinityLab Poroshell 120 EC-C18 3.0 × 150 mm, 2.7 μm, Agilent Technologies, Santa Clara, CA, USA) and gradient solvent systems of acetonitrile (phase A) and acetic acid (0.4%) (phase B) used as a mobile phase. The gradient conditions are reported in Table 2. The flow rate was 0.75 mL min^−1^, and the injection volume was 10 μL.

The method was validated according to the International Council for Harmonisation (ICH) requirements. To evaluate the linearity, precision, and accuracy, the standard solutions were prepared by dissolving appropriate amounts of ETO and CBD in acetonitrile. During the validation procedure, stock solutions were used (~0.2 mg mL^−1^), which were further diluted in acetonitrile to yield concentrations within the calibration ranges. The tested samples (n = 6) were prepared at concentrations of 1 mg of lyophilized NPs per 1 mL of acetonitrile by dissolving the lyophilized NPs in an ultrasound bath for 10 min, and then filtering through 0.22 μm regenerated cellulose syringe filters.

The DL% and EE% were determined using the following equations [32]:

(2) $$DL\%=\frac{m_{API(i)}}{m_{NPs}}\times 100\%,$$

where *m_API_*_(*i*)_ is the mass of active pharmaceutical ingredients (*API*) incorporated, and *m_NPs_* is the mass of lyophilized ETO+CBD-NPs.

(3) $$EE\%=\frac{{DL\%}_{actual}}{{DL\%}_{theoretical}}\times 100\%,$$

where ${DL\%}_{actual}$ is the actual drug loading and ${DL\%}_{theoretical}$ is the theoretical drug loading.

#### 2.3.6. In Vitro Drug Release Studies

CBD and ETO release studies from NPs were conducted using the dialysis bag diffusion method. Briefly, lyophilized ETO+CBD-NPs, CBD-NPs, or ETO-NPs were suspended in water (at a concentration of 5 mg mL^−1^). The solution was placed into a pre-swelled dialysis bag. The membrane (with a 14,000 Da molecular weight cutoff) was cut in such a way that it could accommodate 1.0 mL redispersed nanoformulations sealed at both ends. The dialysis bag with a solution was then immersed in 30 mL of 70% ethanol and incubated at 37 °C under mechanical stirring at 150 rpm. At predetermined time intervals, an aliquot of 1.0 mL was withdrawn and replaced with an equal volume of fresh dissolution medium to ensure sink conditions. The study of API release from individual nanoformulations was carried out in triplicate. The samples were analyzed by HPLC, and the API content was calculated from the average of the standard solution (i.e., the known amount of API in 70% ethanol).

#### 2.3.7. Cellular Uptake

U-138 MG glioblastoma cells were seeded in a six-well plate at a density of 2 × 10^5^ cells per well and were allowed to grow for 24 h. Thereafter, the cells were treated with free substances (ETO and CBD dissolved in 1 mL of DMSO and then diluted with growing medium) or PLGA NPs with ETO and CBD (nanoparticles resuspended in 1 mL of water and then diluted with growing medium) at concentrations of 75 μM CBD and 10 μM ETO, after which they were incubated at 37 °C for 2 h. The control cells were treated with a growing medium containing 0.1% DMSO. After incubation, cells were scraped, washed twice with ice-cold PBS, and collected by centrifugation (4 °C, 4000× *g*). The cell pellets were resuspended in 500 μL of the HPLC mobile phase (acetonitrile and 0.4% acetic acid (60:40 *v*/*v*)) and disrupted by an ultrasonic cell disruptor (Bioruptor Next Generation UCD300, Diagenode, Belgium) for 1 min. Then, the samples were centrifuged for 10 min (4 °C, 15,000× *g*), and 400 μL of the supernatant was filtered and analyzed by HPLC. The intracellular drug content was normalized to the cellular protein content of each plate using the Lowry method.

### 2.4. Statistical Analysis

The data were analyzed using Statistica 12 software (StatSoft). Analysis of variance (ANOVA) was used to determine the statistical significance between samples. The a priori level of significance was *p* < 0.05. Each experiment was conducted in triplicate, and the results were represented as the mean ± standard deviation (SD).

## 3. Results and Discussion

### 3.1. CBD and Its Combination with ETO Reduce the Viability of GBM Cells

**Figure 1:**
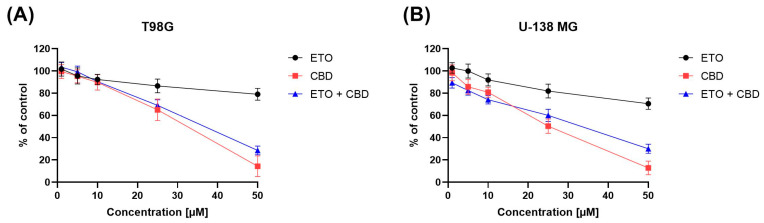
The impact of ETO, CBD, and the combination of ETO and CBD on the cytotoxicity in T98G (**A**) and U-138 MG (**B**) cell lines, measured using a 24 h MTT test. DMSO-treated cells were considered as the control group, with 100% cell viability. The presented values represent the mean ± SEM from three separate experiments, with four measurements per assay.

The impact of ETO, CBD, and their combination on the viability of T98G and U-138 MG cells was evaluated using the MTT assay. Within the concentration range 1–50 μM, CBD, ETO, and their combination reduced the viability of both tested cell lines in a dose-dependent manner (Figure 1). CBD and the combination of CBD and ETO exhibited more potent cytotoxic activity against GBM cell lines as compared to ETO.

The anticancer effect of CBD in glioma cells is, to some extent, facilitated through the activation of endocannabinoid receptors, which subsequently initiate diverse cellular signaling pathways, including the endoplasmic reticulum (ER) stress pathway [33]. Our results are in line with the pioneering work of Massi et al., who reported the antitumor effects of CBD on glioma cell lines [34]. Also, Gross et al. recently demonstrated the cytotoxic nature of CBD in human and canine glioma cells. They suggested a possible mechanism of action of CBD involving the dysregulation of calcium homeostasis and mitochondrial activity [35]. Other cannabinoid-receptor-independent mechanisms are also known to be responsible for the cytotoxic and anti-migratory effects of CBD in GBM cells [36].

In our previous study, we showed moderate cytotoxicity of ETO after 48 h treatment of GBM cells [21]. In this study, we provide evidence that, after 24 h, the cytotoxic effect of ETO is also noticeable at concentrations of 25 μM and 50 μM. In the recent study of Md et al., an ETO-loaded nanoemulsion showed substantially potent cytotoxic effect against lung cancer cells [15]. However, the use of ETO as an adjuvant in standard anti-GBM treatment still requires further investigation.

Moreover, in our study, we also found a similar (as compared to pure CBD) antitumor effect when the combination of CBD and ETO was used. To the best of our knowledge, this is the first study showing the cytotoxic effect of this combination of drugs on GBM cells.

Additionally, the literature provides evidence that the combination of cannabinoids with canonical chemotherapeutics, such as TMZ, potentiates their anticancer effects [37,38]. Thus, additional studies of the combination of TMZ with CBD and ETO are needed to elucidate their potential synergistic effects.

### 3.2. Exposure to CBD and Its Combination with ETO Induces Apoptotic Death of GBM Cells

**Figure 2:**
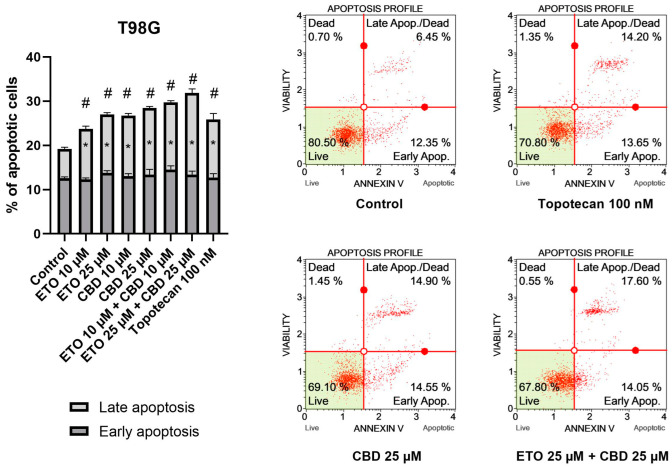
The impact of ETO, CBD, and their combinations on apoptosis in the T98G cell line after 24 h of treatment: The data are presented as the mean ± SEM, derived from two independent experiments; (*) denotes statistically significant differences from the control group for early/late apoptosis, while (#) above the bar indicates statistically significant differences from the control group for total apoptotic cells, with *p* < 0.05. Representative histograms show the results obtained for control (DMSO-treated cells—negative control), 100 nM topotecan (positive control), 25 μM CBD, and the combination of 25 μM ETO and 25 μM CBD. Live cells are shown in the lower-left quadrant of the scatterplot, whereas early-apoptotic cells and late-apoptotic cells/dead cells are shown in the lower-right and upper-right quadrants of the scatterplot, respectively. Dead cells are presented in the upper-left quadrant of the scatterplot.

The results of our study revealed that ETO and CBD, used both as single compounds and in combination, induced apoptotic cell death in GBM cells after 24 h of incubation. In regard to the T98G cell line (Figure 2), the strongest pro-apoptotic effect—namely, 31.89 ± 0.21% apoptotic cells—was exerted by the combination of ETO and CBD, both at concentrations of 25 μM. However, the lower tested concentration, i.e., 10 μM ETO and 10 μM CBD, was also very effective in inducing apoptotic cell death—the observed effect was even stronger than that of 100 nM topotecan, used as a positive control in this assay. Moreover, the individual compounds also increased the percentage of apoptotic cells, both when calculated as a sum of early- and late-apoptotic cells, and in the fraction of only early-apoptotic cells.

**Figure 3:**
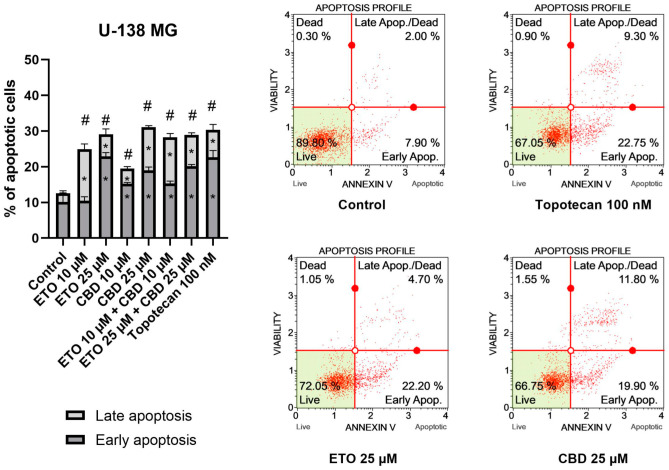
The impact of ETO, CBD, and their combinations on apoptosis in the U-138 MG cell line following 24 h of treatment: The data are expressed as the mean ± SEM, derived from two separate experiments; (*) indicates statistically significant differences compared to the control group for early/late apoptosis, while (#) above the bar indicates statistically significant differences compared to the control group for total apoptotic cells, with *p* < 0.05. Representative histograms show the results obtained for control (DMSO-treated cells—negative control), 100 nM topotecan (positive control), 25 μM ETO, and 25 μM CBD. Live cells are shown in the lower-left quadrant of the scatterplot, whereas early-apoptotic cells and late-apoptotic cells/dead cells are shown in the lower-right and upper-right quadrants of the scatterplot, respectively. Dead cells are presented in the upper-left quadrant of the scatterplot.

The results obtained for U-138 MG cells (Figure 3) resembled those of T98G cells, in that ETO, CBD, and their combination significantly increased the percentage of apoptotic cells. However, in this case, the most pronounced effects were not observed for the mixtures of ETO and CBD, but for the individual compounds. The 24 h exposure to 25 μM CBD resulted in 31.08 ± 1.03% apoptotic cells, whereas 25 μM ETO treatment led to 29.12 ± 2.48% apoptotic cells. The results obtained for the mixtures at both concentrations were also significantly different compared to the DMSO-treated control.

Our results regarding the use of the individual compounds, i.e., CBD and ETO separately, are in line with the currently published reports. In a study by Gross et al., the pro-apoptotic effect of CBD on glioma cell lines was demonstrated [35]. Also, ETO was found to induce apoptotic cell death in A-172 and T98G cell lines [21]. However, to the best of our knowledge, this is the first report showing the increased pro-apoptotic effects of the combination of both compounds in the T98G cell line. Further studies should explore this drug combination, while also including the traditional modes of treatment, i.e., chemo- and radiotherapy. Interestingly, data presented by Ivanow et al. show that the induction of apoptosis was increased after combined treatment of the U87MG and U118MG GBM cell lines with 20 μM CBD and 5 Gy gamma irradiation [39]. Also, wider concentration ranges of those two compounds should be elucidated, as it was shown that CBD, even at low concentrations that are insufficient to induce apoptosis, downregulates the expression of MMP-2, MMP-9, TIMP-4, TIMP-1, and other proteins associated with glioma tumor migration [40,41]. These data provide strong support for further exploration of CBD and ETO in anti-GBM treatment.

### 3.3. CBD and ETO Affect the Distribution of the Cell-Cycle Phases in GBM Cells

**Figure 4:**
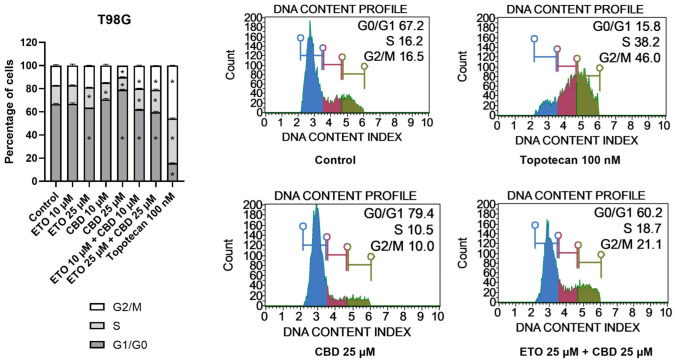
The influence of ETO, CBD, and their combination on cell-cycle distribution in the T98G cell line: The flow cytometry analysis of propidium-iodide- and RNase-A-stained cells allowed for the determination of the percentages of cells in the G1/G0, S, and G2/M phases. Topotecan served as the positive control in this assay. The values presented in the bar chart represent the mean ± SEM obtained from three independent experiments; (*) indicates statistically significant differences from the control group for a specific cell-cycle phase, with *p* < 0.05. On the right-hand side of the bar chart, representative histograms of DMSO-treated cells (referred to as ‘Control’), topotecan (positive control), and the two most active compounds/combinations are provided. The histograms are color-coded by cell-cycle phase: blue indicates G0/G1, purple represents S phase, and green shows G2/M phases.

The examination of the cell-cycle distribution in the T98G cell line (Figure 4) demonstrated that the tested compounds induced changes in the distribution of the cell-cycle phases after 24 h of incubation. The most significant alterations were observed following treatment with 25 μM CBD, which led to an increase in the percentage of cells in the G1/G0 phase, accompanied by a reduction in the numbers of cells in the S and G2/M phases. The lower analyzed concentration of CBD, i.e., 10 μM CBD, exhibited similar effects. However, the results of ETO and its mixture with CBD revealed contrasting results. We observed a slight (though still significant) decrease in the percentage of cells in the G1/G0 phase, accompanied by increases in the amounts of cells in the S and G2/M phases. Similar (though much more pronounced) changes in the cell-cycle distribution were observed in case of 100 nM topotecan.

**Figure 5:**
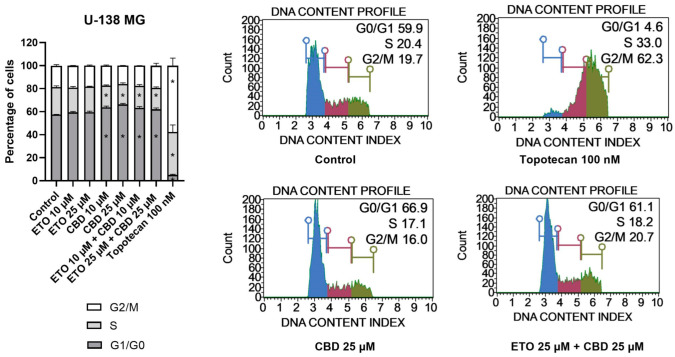
The impact of ETO, CBD, and their combination on cell-cycle distribution in the U-138 MG cell line: The percentages of cells in the G1/G0, S, and G2/M phases were analyzed by flow cytometry after staining with propidium iodide and RNase A. Topotecan was used as a positive control in this assay. The values on the bar chart are shown as the mean ± SEM calculated from three independent experiments; (*) indicates statistically significant differences from the control group for a particular phase, with *p* < 0.05. Representative histograms of the DMSO-treated cells (designated as ‘Control’) and topotecan (positive control), as well as the two most active compounds/combinations, are shown on the right-hand side of the bar chart. The histograms are color-coded by cell-cycle phase: blue indicates G0/G1, purple represents S phase, and green shows G2/M phases.

With regard to U-138 MG cells (Figure 5), we did not observe any changes in the cell-cycle distribution when the cells were incubated with ETO, whether at concentrations of 10 μM or 25 μM. The impact of CBD, however, was significant. Similarly, as in the T98G cell line, CBD increased the number of G1/G0 cells while lowering the numbers of cells in the S and G2/M phases of the cell cycle. Analogous but slightly less pronounced changes were observed for the mixture of CBD with ETO. The most pronounced changes in the cell-cycle distribution were observed for 100 nM topotecan.

To conclude, here we showed the potential of CBD and the combination of CBD and ETO to induce cell-cycle arrest in U-138 MG and T98G cells, respectively. The literature also provides evidence of similar effects in other cancers. In this regard, Morelli et al. reported that CBD induced cell-cycle arrest in TRPV2-transfected multiple myeloma cells through activation of the NF-κB pathway, the elevation of reactive oxygen species, reduction in cyclin D1, and the inhibition of ERK activation [42]. Zhang et al. showed that CBD could induce G0–G1-phase cell-cycle arrest and apoptosis by increasing ROS production, leading to the inhibition of gastric cancer cell proliferation [43]. An ETO-loaded nanoemulsion was also shown to improve the anticancer activities against lung cancer cells. Similar studies regarding GBM cells are lacking [15]. Thus, in order to fulfill the need for the evaluation of CBD and ETO nanoformulations in the treatment of GBM, we developed PLGA-based NPs loaded with an ETO–CBD combo.

### 3.4. Development and Physical Characterization of PLGA-Based NPs Loaded with an ETO–CBD Combo

**Figure 6:**
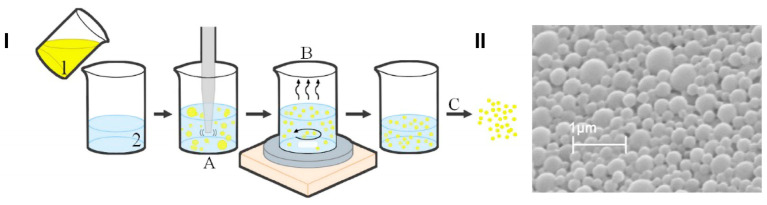
Scheme of the preparation method of PLGA-based NPs loaded with **the** ETO–CBD combo (**I**); scanning electron microscopy microphotograph of ETO+CBD-NPs (**II**); 1—organic phase (ETO, CBD, and PLGA dissolved in dichloromethane), 2—aqueous phase (2% PVA), A—homogenization, B—solvent evaporation, C—lyophilization.

In this work, we developed and characterized ETO and CBD co-loaded PLGA NPs. The co-entrapment of two drugs that differ in their physicochemical properties (such as lipophilicity and molecular weight) into NPs is a challenging task and requires optimization of the preparation method to obtain high EE% and optimal size and surface charge sufficient for delivery to the brain. The PLGA NPs developed using the emulsification and solvent evaporation method exhibited optimal physicochemical properties for the co-delivery of the studied substances (Figure 6). Scanning electron microscopy microphotographs confirmed the spherical shape and smooth surface of the developed NPs. They were characterized by an MPS of 397 nm and a negative charge (ZP of −13.2 mV). Comparing ETO+CBD-NPs to blank-NPs, ETO-NPs, and CBD-NPs, it was found that the MPS of NPs loaded with both drugs was significantly higher than that in the formulations without drugs or loaded with only one of them. Similarly, the PDI was the highest for the ETO+CBD co-loaded formulation. In the case of ZP, higher negative values were found for the loaded NPs than for the blank ones. However, when comparing co-loaded NPs with NPs loaded with only one substance, the absolute value of ZP was lower (Table 3).

Fraguas-Sánchez et al. obtained PLGA NPs loaded with CBD, which were characterized by an MPS of 236 ± 12, a negative ZP (−16.6 ± 1.2), and a PDI value equal to 0.165 ± 0.009 [44]. In another study concerning the loading of *Cymbopogon citratus* essential oil into PLGA NPs, the following results were obtained: MPS of 277.0 ± 5.5 nm, PDI of 0.18 ± 0.04, and ZP of −16.1 ± 1.8 mV [45]. Moreover, in the curcumin-loaded PLGA NPs studied by Chen et al. [46], the MPS value was 327.9 ± 14.5 nm, the PDI was lower than 0.21, and the ZP was −14.8 ± 1.6 mV. The results obtained in our study are comparable with the values found in the literature.

**Figure 7:**
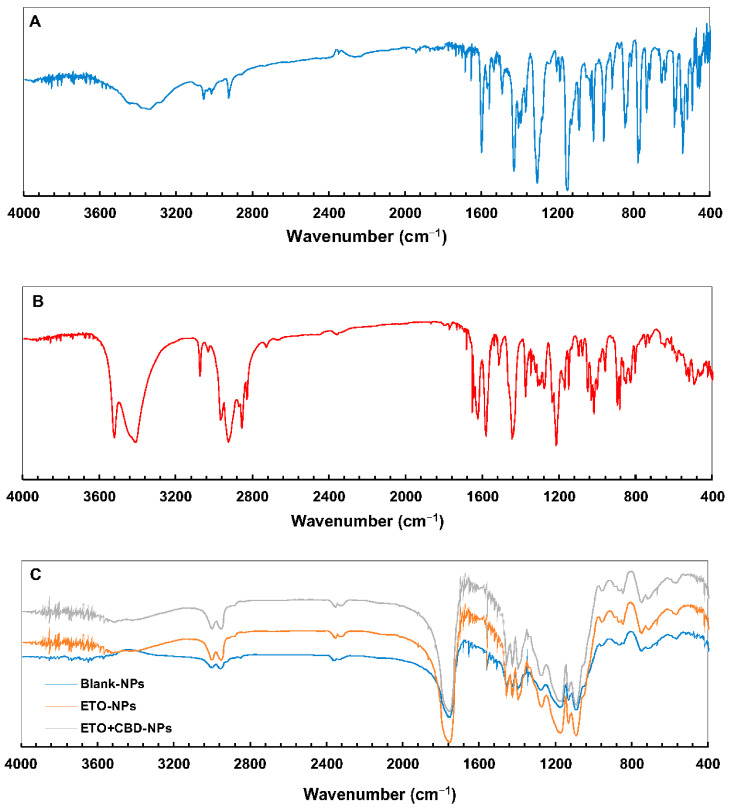
FT-IR spectra of ETO (**A**), CBD (**B**), and PLGA NPs (**C**).

The PLGA FT-IR spectrum (Figure 7) showed characteristic vibrations of the C–H group at the wavelengths of 2953 and 2999 cm^−1^, and an intense peak at the wavelength of 1751 cm^−1^ corresponded to the vibrations of the C=O group. In the ETO FT-IR spectrum, there were absorption bands for −C=N at 1147, 1305, and 1427 cm^−1^, characteristic of vibrations of –S=O bonds and 653, 777, and 846 cm^−1^, which corresponded to–C–Cl stretching vibrations. For CBD, the characteristic vibrations in the FT-IR spectrum were observed at 3525 cm^−1^ and 3431 cm^−1^ (O–H, stretching vibrations), 2931 cm^−1^ and 2856 cm^−1^ (C–H, stretching vibrations), 1625 cm^−1^ and 1585 cm^−1^ (aromatic C=C, stretching vibrations), and 1446 cm^−1^ (C–H, deformation vibrations).

The FT-IR spectra of ETO-NPs and ETO+CBD-NPs showed great similarity with the spectrum of the unloaded formulation, which indicates that both substances were enclosed in the polymeric matrix. The main absorption bands of ETO and CBD in the spectra of their NPs did not appear or were of very low intensity, indicating that there was an interaction between both drugs (ETO and CBD) and PLGA. Comparison of the spectra of the ETO-NPs, CBD-NPs, and ETO+CBD-NPs with the unloaded NPs (blank-NPs), ETO, and CBD spectra showed that the spectra of the loaded NPs were correlated with the blank-NPs spectrum. The calculated purity index was in the range of 0.9943–0.9983.

**Figure 8:**
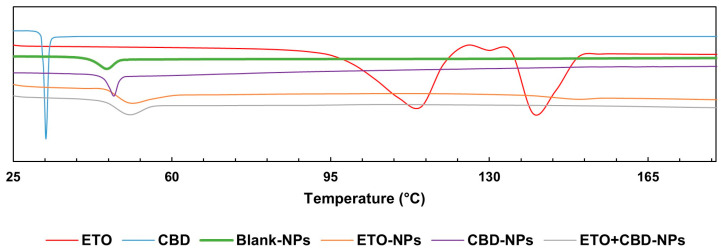
DSC endotherms for ETO, CBD, and PLGA NPs.

The DSC curve of CBD was characterized by a single endothermic peak that appeared around the compound’s melting point (32 °C). The DSC thermogram of ETO was characterized by an endothermic peak at 114 °C indicating the glass transition temperature, a peak at 124 °C corresponding to recrystallization, and a peak at 139 °C to which melting could be attributed. In the case of PLGA, a peak was observed at approximately 45 °C, corresponding to the polymer’s glass transition temperature. The FT-IR and DSC (Figure 8) results showed that ETO and CBD were dissolved in the polymeric matrix and no drug residuals remained on the surface of the NPs. The same methodology was applied by Fraguas-Sánchez et al. to confirm the successful entrapment of CBD in NPs [44].

### 3.5. DL%, EE%, and In Vitro Drug Release

A new HPLC method was developed and validated for simultaneously determining ETO and CBD. This method was used to determine DL%, EE%, and the drug release profile. The selectivity of the HPLC method was evaluated by verifying the chromatograms of CBD (t_R_ = 6.44 min), ETO (t_R_ = 1.76 min), and blank samples (acetonitrile and PLGA solution t_R_ < 1.12 min). The linearities were established in the ranges of 0.10–70.81 μg mL^−1^ for CBD (r = 0.9999) and 0.10–70.56 μg mL^−1^ for ETO (r = 0.9999). The limits of detection were 0.03 μg mL^−1^ and 0.02 μg mL^−1^ and the limits of quantitation were 0.08 μg mL^−1^ and 0.06 μg mL^−1^ for CBD and ETO, respectively. The precision and accuracy studies were carried out for CBD and ETO at two concentrations, each with six samples, in two series (each series was conducted with intervals of a few days). The precision of the method was estimated in relation to repeatability (intraday) and intermediate precision (interday). The values of relative standard deviation expressed in percentages (RSD%) for the repeatability and intermediate precision were found to be within acceptable limits (Table 4). The accuracy of the method (intra- and interday) was determined on the basis of recovery, expressed as percentages (the comparison of the experimental concentration to the expected real concentration). The average percentage recoveries (between 98.01% and 101.85%) showed satisfactory accuracy (Table 4).

The EE% and DL% of ETO and CBD in co-loaded NPs were determined for six independent samples obtained in separate production series. The mean values of the EE% and DL% of ETO+CBD-NPs were equal to 10.94 ± 1.79% and 0.50 ± 0.07% for ETO, respectively, and 78.43 ± 5.43% and 3.5 ± 0.3% for CBD, respectively. The lower EE% of ETO was associated with higher hydrophilicity of ETO (logP = 3.3) compared to CBD (logP = 6.5) and, thus, the higher solubility and affinity for the external phase of the emulsion of PLGA in a 2% solution of PVA obtained in the course of the NPs’ preparation. The obtained ETO+CBD-NPs are the first delivery systems developed for the combination of ETO and CBD. Our results showed that the physicochemical properties of NPs are sufficient for the further development of intravenous dosage forms to be used in the treatment of glioblastoma.

Release studies in sink conditions are performed routinely for newly developed formulations to compare them with one another and to investigate their ability to release the drug. A particular case is nanoformulations, which are often used as a delivery system for poorly soluble active compounds, which cause many difficulties in this type of study [47].

The conventional dissolution test using USP apparatus I and II with the preservation of sink conditions is challenging to maintain for nanoformulations containing poorly soluble active compounds. Such substances need a large volume of release medium, which may make it difficult or impossible to determine the content of the released API due to the critically low concentration. On the other hand, the small size of the nanoformulation makes it challenging to separate the NPs from the medium. This requires an appropriate procedure (e.g., high-speed centrifugation for sample collection, or the use of a dialysis bag). The literature review showed that, due to the above difficulties, studies on API release from nanoformulations are often carried out in suboptimal conditions, without ensuring sink conditions [47].

In order to ensure sink conditions, a flow-through apparatus can be used, or appropriate modifiers can be added to the release solution, e.g., surfactants, inorganic salts, and organic co-solvents. An alternative method may be biphasic dissolution systems, where the dissolution medium contains immiscible aqueous and organic layers. However, each of the above options has its limitations. For example, adding a surfactant may cause micelles to form, which may prevent the released API from passing through the dialysis membrane. On the other hand, the addition of organic solvents is not physiologically justified [47,48].

The review of articles reporting studies concerning the in vitro release kinetics of APIs from NPs showed that various solutions can be applied as the release medium for nanoformulations containing poorly soluble compounds. Examples include a mixture of water or buffer (usually PBS) and a surfactant, e.g., Tween 80, sodium dodecyl sulfate, poloxamer 188, and/or ethanol (in concentrations ranging from a few percent to 70%), as well as 0.9% NaCl or serum-containing medium [45,46,48,49,50,51,52,53].

**Figure 9:**
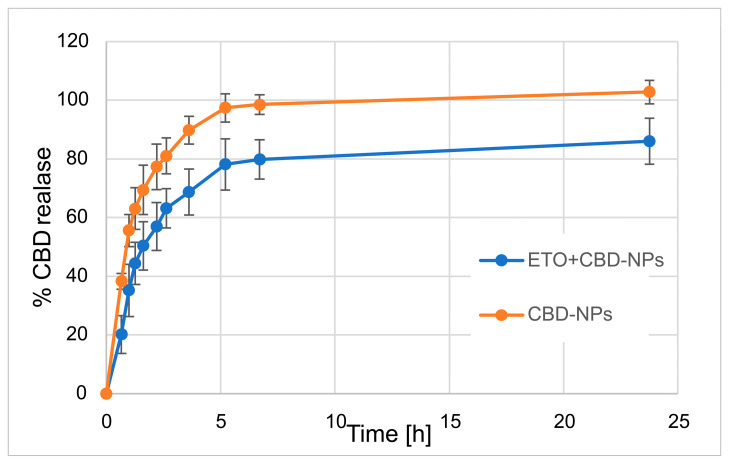
Release profile of CBD from CBD-NPs and ETO+CBD-NPs.

**Figure 10:**
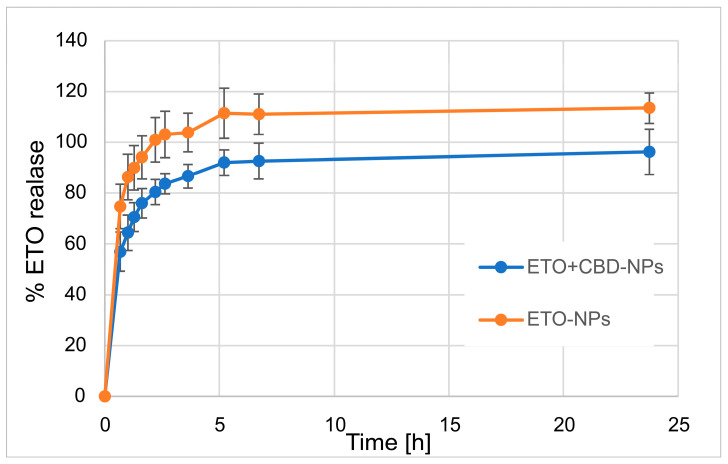
The release profile of ETO from ETO-NPs and ETO+CBD-NPs.

In our work, we focused on comparing the release profiles of APIs from the obtained nanoformulations containing only one active ingredient (ETO or CBD) with those from nanoformulations with both compounds (CBD-ETO-NPs). We used the dialysis bag technique and 70% ethanol as the medium for the above studies. Although the experimental conditions did not simulate physiological conditions, they enabled the comparison of the obtained formulations and ensured the solubility of the released drugs at the appropriate level. The obtained data were expressed as the percentage of cumulative API release versus time and are presented in Figure 9 and Figure 10. The shapes of the obtained release profiles were similar. The release behavior of the APIs (especially ETO) from the tested PLGA NPs initially showed a high burst effect, which is likely explained by a rapid release of the drug incorporated in the surface part of the NPs, followed by a slower one (to reach a plateau at about 100%) connected with the release of the API(s) incorporated inside the NPs [30]. A similar profile shape was observed, among others, when releasing R-flurbiprofen from PLGA NPs in a PBS medium [52] or doxorubicin in 0.9% NaCl [53].

However, it should be noted that, in the case of ETO-NPs and CBD-NPs, the percentages of ETO or CBD released simultaneously were significantly higher than that obtained with the formulations prepared with both active compounds. Complete release (near 100%) of ETO was observed after 2.2 h (100.9 ± 8.7%) and 24 h (96.2 ± 8.9%) for ETO-NPs and ETO+CBD-NPs, respectively. In the case of CBD-NPs, the complete release was observed after 7 h (98.5 ± 3.3%), but for ETO+CBD-NPs, after 24 h, the release percentage was 86 ± 7.8%. Based on the above results, another conclusion can be drawn: the ETO release process from the NPs is faster than the release of CBD, which may be related to their different lipophilicity.

### 3.6. Uptake of ETO+CBD-NPs by U-138 MG Cells

**Figure 11:**
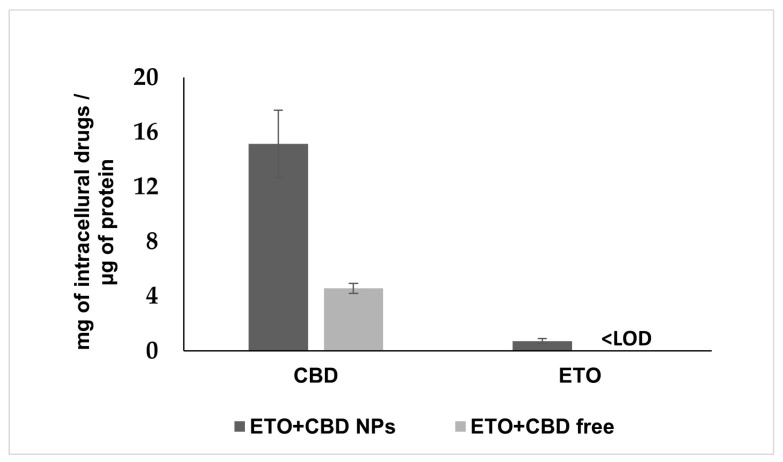
Cellular uptake of ETO and CBD from the DMSO solution (ETO+CBD free) and ETO+CBD-NPs. Results are shown as the mean ± SD.

PLGA NPs are well-known carriers and efficient delivery vehicles. Therefore, we examined whether NPs more effectively delivered ETO and CBD into the cancer cells as compared to their free forms. To determine this, we treated the U-138 MG cells either with ETO+CBD-NPs or with free ETO and CBD for 2 h. Subsequently, intracellular accumulation of both drugs was quantified by the HPLC method in cell lysates (Figure 11). The data showed that the intracellular accumulation of ETO and CBD in cancer cells treated with NPs was significantly higher (CBD, 3.31-fold) as compared to those treated with free substances. The intracellular concentration of ETO in cells treated with free substances was found to be lower than the limit of detection of the HPLC method used (0.02 μg mL^−1^).

Glioblastoma cells are able to absorb both drugs, since their synergistic effect was shown in this study. However, the appropriate formulation may increase their uptake through the membrane, enhancing the cytotoxic effect. Our results suggest that NPs enhance the cytotoxic potential of ETO and CBD due to their efficient delivery to tumor cells. After 2 h of incubation, the intracellular concentration of CBD was 3.31-fold higher as compared to the assay where free substances were used. In the case of ETO, we could detect the intracellular substance only when it was delivered using NPs; in the case of the free substance, the drug concentration was below the HPLC method’s limit of detection. Similarly, delivery of PLGA NPs to glioblastoma cells was also studied for paclitaxel/methotrexate co-loaded PLGA NPs coated with PVA/P188 [51] and paclitaxel and R-flurbiprofen D-α-tocopherol polyethylene glycol succinate-coated PLGA NPs [52]. These studies suggest that PLGA NPs’ coating may additionally enhance their cellular uptake.

PLGA NPs have already been investigated for glioblastoma drug delivery. Ramalho et al. developed PLGA-based NPs for co-delivery of temozolomide and O6-benzylguanine [54]. Maliyakkal et al. showed that cisplatin loaded into PLGA NPs exhibited augmented drug accumulations and inhibition of multidrug-resistance transporters in human glioblastoma cells [55]. Maksimenko et al. optimized doxorubicin-loaded PLGA NPs coated with poloxamer 188. Such a delivery system enabled the delivery of doxorubicin to the brain, which normally cannot penetrate across the blood–brain barrier in its free form [53]. Similar formulations have been developed for methotrexate and paclitaxel. Both of these drugs were co-loaded into PLGA NPs that were further coated with polyvinyl alcohol and Poloxamer 188. The blood compatibility and glioblastoma cellular uptake of these NPs were confirmed [51]. Caban-Toktas et al. developed positively charged chitosan-modified PLGA NPs for the combination of paclitaxel and R-flurbiprofen to target glioblastoma tumor cells [52]. PLGA NPs can be easily functionalized with different materials [50]; therefore, the obtained NPs co-loaded with ETO and CBD can be subjected to further modification with various targeting moieties and, thus, are promising formulations for further investigations.

## 4. Conclusions

This study aimed to investigate the synergistic antitumor activity of ETO and CBD in a model of GBM cell lines, and to develop PLGA-based NPs co-loaded with ETO and CBD. In the tested GBM cell lines (T98G and U-138 MG), the resulting combination of ETO and CBD reduced cell viability in a dose-dependent manner and induced apoptosis. NPs based on poly(lactic-co-glycolic acid) were obtained using the emulsification and solvent evaporation method. Their physicochemical properties (such as shape, size, EE%, and quality characteristics) were determined. The combination of ETO and CBD may be a promising adjuvant therapy for treating GBM, and the effective incorporation of these compounds in nanoformulations allows for the further development of pharmaceutical formulations.

## Figures and Tables

**Table 1:** Compositions of the developed PLGA NPs.

Formula	PLGA (mg)	ETO (mg)	CBD (mg)	PVA (%, *w/v*)
Blank-NPs	50	-	-	2
CBD-NPs	50	-	2.5	2
ETO-NPs	50	5	-	2
ETO+CBD-NPs	50	2.5	2.5	2

**Table 2:** HPLC gradient used for the determination of CBD and ETO.

Time (min)	Phase A (%)	Phase B (%)
0	40	60
2	40	60
4	90	10
10	90	10
11	40	60
15	40	60

**Table 3:** Characterization of the studied PLGA NPs.

Formula	MPS ± SD (nm)	PDI ± SD	ZP ± SD (mV)
Blank-NPs	245 ± 2.1	0.029 ± 0.009	−11.5 ± 0.26
ETO-NPs	319 ± 24	0.145 ± 0.017	−15.1 ± 0.12
CBD-NPs	225 ± 4.4	0.038 ± 0.003	−17.4 ± 0.70
ETO+CBD-NPs	397 ± 6.2	0.256 ± 0.044	−13.2 ± 0.15

**Table 4:** The precision and accuracy of the method of determining CBD and ETO.

Precision/Recovery					
	Intraday				Interday
	Series I n = 6		Series II n = 6		Series I-II n = 12
	Concentration (μg mL^−1^)	RSD (%) Recovery (%)	Concentration (μg mL^−1^)	RSD (%) Recovery (%)	RSD (%) Recovery (%)
CBD	10.12	1.59 98.01 ± 1.64	10.11	1.06 99.28 ± 1.11	1.45 98.64 ± 0.91
	55.63	0.73 100.17 ± 0.77	55.61	1.53 99.49 ± 1.60	1.21 99.83 ± 3.78
ETO	10.06	0.39 101.61 ± 0.41	10.09	1.89 101.85 ± 2.02	1.31 101.73 ± 0.85
	55.44	0.58 99.85 ± 0.60	55.33	0.05 99.04 ± 0.05	0.58 99.44 ± 1.83

## Data Availability

The data presented in this study are available on request from the authors.
